# Protection of Residual Renal Function and Nutritional Treatment: First Step Strategy for Reduction of Uremic Toxins in End-Stage Kidney Disease Patients

**DOI:** 10.3390/toxins13040289

**Published:** 2021-04-19

**Authors:** Adamasco Cupisti, Piergiorgio Bolasco, Claudia D’Alessandro, Domenico Giannese, Alice Sabatino, Enrico Fiaccadori

**Affiliations:** 1 Department of Clinical and Experimental Medicine, University of Pisa, 56121 Pisa, Italy; dalessandroclaudia@gmail.com (C.D.); domenico.giannese@phd.unipi.it (D.G.); 2 “Conservative Treatment of Chronic Kidney Disease” Project Group of the Italian Society of Nephrology, 00185 Rome, Italy; pg.bolasco@gmail.com; 3 Department of Medicine and Surgery, University of Parma, Nephrology Unit, Parma University Hospital, 43121 Parma, Italy; alice.sabatino86@gmail.com (A.S.); enrico.fiaccadori@unipr.it (E.F.)

**Keywords:** uremic toxins, nutrition, infrequent dialysis, dietary protein, nutritional therapy, residual kidney function, CKD, ESKD

## Abstract

The retention of uremic toxins and their pathological effects occurs in the advanced phases of chronic kidney disease (CKD), mainly in stage 5, when the implementation of conventional thrice-weekly hemodialysis is the prevalent and life-saving treatment. However, the start of hemodialysis is associated with both an acceleration of the loss of residual kidney function (RKF) and the shift to an increased intake of proteins, which are precursors of uremic toxins. In this phase, hemodialysis treatment is the only way to remove toxins from the body, but it can be largely inefficient in the case of high molecular weight and/or protein-bound molecules. Instead, even very low levels of RKF are crucial for uremic toxins excretion, which in most cases are protein-derived waste products generated by the intestinal microbiota. Protection of RKF can be obtained even in patients with end-stage kidney disease (ESKD) by a gradual and soft shift to kidney replacement therapy (KRT), for example by combining a once-a-week hemodialysis program with a low or very low-protein diet on the extra-dialysis days. This approach could represent a tailored strategy aimed at limiting the retention of both inorganic and organic toxins. In this paper, we discuss the combination of upstream (i.e., reduced production) and downstream (i.e., increased removal) strategies to reduce the concentration of uremic toxins in patients with ESKD during the transition phase from pure conservative management to full hemodialysis treatment.

## 1. Introduction 

In end-stage kidney disease (ESKD), progressive retention of molecules which are normally excreted into urine occurs, causing impairment of several biological functions. These substances are known as uremic toxins [1]. They are quite different regarding their physicochemical aspects, the substrates from which they derive, the pathways of generation, and the routes of removal from the body. Uremic toxins contribute to clinical manifestations of the uremic syndrome, together with the changes in hormonal status, water, and electrolyte homeostasis as well as in mineral metabolism [1].

**Figure 1:**
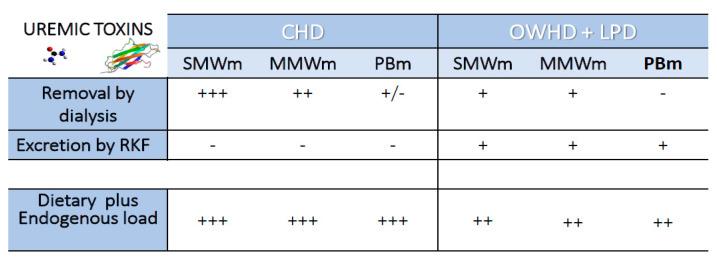
Theoretical comparison of production and removal rates of uremic toxins between conventional hemodialysis (CHD) and once-a-week hemodialysis combined with a low-protein diet (OWHD + LPD). LPD: standard low-protein diet; RKF Residual Kidney Function; SMWm: Small molecular weight molecules; MMWm: medium molecular weight molecules; PBm protein-bound molecules; +++ elevate; ++ moderate; + mild; - null.

Although this definition also includes inorganic molecules, namely potassium, sodium, hydrogen ions, and phosphates, the term uremic toxins usually refers to organic molecules in nature. Organic uremic solutes mainly derive from the catabolism of exogenous animal protein, both directly and indirectly. The European Uremic Toxin Work Group (EuTox) identified 90 organic compounds that may be classified by their molecular mass, and by their grade of water solubility and/or bounding to circulating proteins [2]. Both the molecular weight (MW) and the protein binding capacity of uremic compounds are clinically important, since they significantly influence the chance of removal by hemodialysis, which is very high for non-protein-bound and low MW molecules (<500 D), but very scarce for protein-bound and high MW (>12.000 D) compounds [3]. These latter can be eliminated only by native kidneys, and this underlines the important role of residual kidney function (RKF) [4] (Figure 1).

Reduction of renal excretion by impaired kidney function is the main mechanism causing the increased serum concentration of uremic solutes, but enhanced synthesis or decreased degradation may occur as well. Serum urea is considered as a marker of nitrogen retention, but it is not the only or the most important one [5]. Retained nitrogen compounds originate mainly from dietary proteins; hence, dietary intake must be considered as the most important source of substrates from which uremic toxins can originate. Both the amount and the quality of protein intake (plant- or animal-based) are important determinants of uremic solutes production, so that a low-protein diet is the mainstay of nutritional therapy of advanced CKD [6,7,8,9].

Uremic solutes can be defined uremic toxins when their increased levels are associated with clinical manifestations. Symptoms related to the retention of uremic toxins are usually more severe in stage 5 CKD, even though their retention and metabolic action may begin at earlier stages of kidney dysfunction.

Considering the well know limitations of the dialysis procedure, it cannot be relied upon as the only approach for the correction of uremic intoxication. Several other strategies to reducing uremic intoxication have been proposed, as both increasing dialysis removal and reducing intestinal uptake. Namely, dialysis removal of protein-bound uremic toxins can be increased by using protein-bound competitors, for example ibuprofen [10]. Some in vitro hemodiafiltration studies also suggest that protein protein-bound uremic solutes are removed more efficiently in the presence of increased ionic strength [11]. At the intestinal level, possible options are of adsorbents of uremic toxins; among them, several data exist about active charcoal AST-120 [12]. When possible, limiting the exogenous sources of uremic toxins by reducing the intestinal absorption and/or decreasing their intestinal production could represent further effective strategies, in parallel to careful protection of the RKF [13].

In this regard, the reduction of dietary intake of animal proteins in association with the increased intake of plant-based foods on the one hand, and the preservation of the RKF on the other hand, should represent the two major targets in patient management in order to counteracting the retention of several uremic toxins. Recently, attention has also been focused on the intestinal microbiota.

The intestinal ecosystem is formed by both the intestinal microbiota and the gut itself where a collection of microorganisms lives in a dynamic interaction with the host metabolism. Microbial colonization reaches its maximum in the colon, where the oxygen content is minimum [14]. The distal gastrointestinal tract represents a favorable environment for bacterial growth, since it is rich in undigested carbohydrates and proteins, which can be fermented under bacterial anaerobic metabolism [15]. In the presence of adequate amounts of undigested carbohydrates (i.e., dietary fibers), proteins are mostly used for bacterial growth, thus favoring bacterial saccharolytic species. On the other hand, when carbohydrates are scarcely present, proteins are fermented by proteolytic bacteria to produce energy, leading to end-products that include potentially toxic molecules (ammonia, amines, thiols, phenols, and indoles) [16]. ESKD may significantly and negatively affects the structure and function of the intestinal barrier as well as the composition and metabolism of the gut microbiota [17,18].

The presence of CKD/ESKD has been linked to relevant quantitative and qualitative abnormalities of intestinal microbiota, in particular, a shift from saccharolytic bacteria towards an increase in the concentration of proteolytic bacteria [19,20,21]. Intestinal dysbiosis is facilitated by reduced intake of fibers [22], which is likely a consequence of dietary restrictions aimed at reducing potassium and phosphorus intake. In addition, patients with CKD/ESKD seem to have impaired protein digestion and absorption [19], increasing the availability of large amounts of undigested proteins for bacterial fermentation in the colon [23,24]. All these factors combined with others related to the treatment of CKD/ESKD itself (e.g., dialysis modality, use of intestinal binders, antibiotics, etc.) cause constipation and changes in the amount and composition of intestinal microbiota. Another key element in the intestinal dysbiosis of patients with CKD/ESKD is the massive influx of urea in the intestine, which produces ammonia and increases intestinal pH [25]. The gut dysbiosis is of particular concern in this clinical setting because many uremic toxin precursors are produced in the intestinal lumen during protein fermentation (putrefaction) by the proteolytic bacteria. The two most widely studied uremic toxin precursors are p-cresol and indole, generated during the fermentation of the amino acids tyrosine and tryptophan, respectively. The p-cresol and indole are further metabolized by the colon mucosa and by the liver to indoxyl sulfate (IS) and p-cresyl sulfate (PCS). These molecules circulate tightly bound to albumin [26] and have high cellular toxicity, especially in the cardiovascular level [27,28]. In healthy subjects, both IS and PCS are actively excreted by the kidneys. This is the main reason why IS and PCS plasma levels progressively increase along with the reduction of GFR, reaching their maximum concentration in patients with ESKD because of their poor removal by hemodialysis [29]. Moreover, some evidence exists that bacterial generation rates in the gut do not change in the different CKD stages and this makes the loss of RKF the major role for the increased protein-bound uremic toxins levels, such as IS and PCS [30]. Another important gut microbiota-derived uremic toxin is trimethylamine-N-oxide (TMAO), derived from l-carnitine and phosphatidylcholine, being associated with increased mortality and cardiovascular morbidity [31]. It accumulates in the blood of patients on hemodialysis, but differently from IS and PCS, TMAO does not circulate bound to albumin; thus, it is more easily removable by dialysis, though at a lower efficiency than urea [32].

## 2. Protection of Residual Kidney Function

Since RKF protection is a major issue in patients with ESKD, several measures have been recommended in this regard [4,33,34,35].

RKF entails considerable advantages for the patient, in terms of better volume control, mineral and electrolyte homeostasis preservation, and improved nutritional status [36]. In addition, unlike dialysis, RKF allows a more efficient elimination of medium molecular weight and protein-bound molecules, a process occurring slowly but continuously over the entire day. Even a small amount of RKF plays an important role in the catabolism of some cytokines (TNFα and IL-1) and other pro-inflammatory and pro-oxidant molecules [37,38]. A lower inflammatory status was observed in the CHOICE study, where patients with urine output volume of at least 250 mL/day showed reduced levels of C reactive protein and IL-6 when compared to patients with urine volumes below 250 mL [36].

Progressive decline of RKF is more rapid in males, in the presence of proteinuria, diabetes mellitus and uncontrolled arterial hypertension; left ventricular hypertrophy, coronary artery disease and congestive heart failure are prevalent comorbid conditions associated with RKF loss [39,40]. The use of RAAS inhibitors is recommended by the National Kidney Foundation’s Kidney Disease Outcomes Quality Initiative (KDOQI) guidelines because it seems to reduce the risk of RKF loss [41]. Several elements related to dialysis treatment are also associated with the decline of RKF, such as biocompatibility and volume management, rhythm and frequency of dialysis. In fact, despite the use of more biocompatible synthetic dialysis membranes and ultrapure dialysate, a slower and gradual decline of RKF can be witnessed, justified by a reduction in the pro-inflammatory and pro-oxidant stimulus triggered by the intra- and post-dialytic hypercatabolism [33]. Episodes of intradialytic hypotension, often generated by a high ultrafiltration rate, result in repeated ischemic bouts affecting the residual renal parenchyma, causing its progressive functional and structural deterioration. Daugirdas et al., in the Frequent Hemodialysis Network Daily and Nocturnal Trial, reported that daily dialysis (6 days/week) promotes faster loss of RKF than standard dialysis [42]. Patients on thrice-weekly hemodialysis also had a greater loss of RKF than patients on twice-weekly treatment [43]. Hence, evidence exists of a direct relationship between the number of dialysis sessions per week and the RKF loss, leading to the concept that, as a clinical practice strategy for starting dialysis, “less is better.” Preservation of RKF is also a crucial aspect of the incremental approach in peritoneal dialysis, where the small hemodynamic changes, preserving from repeated hypoperfusion stress to the kidney, allow to avoid the acceleration of the loss of RKF that generally occurs in the full-dose hemodialysis schedule. In fact, it is well known that the rate of RKF decline was slower in peritoneal than extracorporeal dialysis patients. Moreover, an incremental approach to dialysis seems to slow down the loss of RKF in respect to full dialysis dose, probably thanks to the lower hemodynamical impact and hypoperfusion challenge on residual nephrons when low ultrafiltration rates are required. This is also true for incremental peritoneal dialysis programs, as shown by Sandrini et al. [44] and by Lee et al. [45]. In the clinical course of a patient with CKD, the stage 5 is characterized by very poor kidney function, and hence, by the maximum chance of toxins retention; thus, particular attention should be paid to the timing and the type of dialysis during the transition phase. The start of a full dialysis schedule (thrice weekly) is associated with a rapid loss of RKF, and it requires a high protein intake to maintain a good nutritional status. Despite its favorable effect on LMW molecules and water-soluble toxins, patients on hemodialysis are prone to an increase in the production of uremic toxins because of the high protein intake and, at the same time, there is a reduction in the clearance of larger and protein-bound molecules (by the loss of RKF and by the low efficiency of hemodialysis removal).

An incremental approach with twice-weekly dialysis resulted to be protective towards RKF [46]. However, in this particular case, the combination of a low-protein diet on non-dialysis days with a high-protein diet on dialysis days may raise some concerns related to dietary adequacy. In fact protein catabolism may also be affected by dietary proteins, with higher intakes leading to increased protein turnover. Low-protein diets in patients on conservative treatment are feasible and nutritionally safe because the nitrogen balance is maintained due to an adaptation of protein turnover following reduced protein intake; in fact, protein and amino acid degradation is reduced and their recycling becomes more efficient [47,48]. Such adaptation is possible only if there is adequate intake of essential amino acids and energy, and if metabolic acidosis is corrected [48]. Some authors believe that in the case of a twice-weekly hemodialysis, in which patients more frequently change the daily amount of protein intake, there would not be enough time to allow the above adaptation in protein turnover [49]. There are also data suggesting a more positive nitrogen balance and better preservation of RKF in patients on peritoneal dialysis receiving low-protein diets supplemented with KA/EES [49]. However, more studies are needed before recommending this approach in patients undergoing twice-weekly hemodialysis.

Conversely, an incremental approach based on once-a-week hemodialysis schedule coupled with low protein intake for the six non-dialysis days, could represent a possible strategy that could potentially preserve RKF while still allowing native kidney toxin removal, while, at the same time, the toxin production from a low (animal) protein load is reduced [50,51]. Since LPDs are associated with reduced phosphorus and sodium intake, and lower inorganic acid production, these balances would be better controlled despite a lower rate of extracorporeal dialysis removal in the infrequent schedules.

## 3. Nutritional Treatment

Since the 1960s, protein intake reduction has been recommended as the mainstay of dietary treatment of CKD, since it reduces nitrogen waste product generation, of which urea is the biochemical marker [52]. In addition to reducing salt and phosphorus intake, low protein intake helps to improve CKD-MBD, volume expansion, and metabolic acidosis, and helps to reduce uremic symptoms [8,19]. Together with these traditional goals of the nutritional treatment in CKD patients, a novel line of research has emerged, focusing on the abnormalities of intestinal microbiota in CKD and the changes induced by dietary treatment [19,20].

Protein restriction is the most important part of the dietary manipulation in CKD patients. It includes a wide spectrum of intakes suggested, from “low protein” diets supplying 0.6–0.7 g/kg/day of proteins, up to “very low-protein diets” (0.3–0.4 g/kg/day) supplemented with essential amino acids and ketoacids. Although protein restriction has a pivotal role, it is only one part of a more complex intervention on CKD patients’ dietary habits. In fact, nutritional recommendations for CKD patients include the restriction of phosphorus and sodium intake, together with an adequate calory intake to cover the energy requirements [53]. These “quantitative” recommendations are accompanied by qualitative tips in regard to the selection of foods, with a preference for those of plant origin characterized by a reduced content of sulfur amino acid and increased contentof fibers, and suggestions on food preparation and cooking, aimed at reducing the mineral content and making dishes more palatable despite the many limitations [53]. Nutritional therapy induces favorable metabolic changes, prevents signs and symptoms of renal failure, and is able to delay the need for dialysis. A low-protein diet is able to reduce the nitrogen load in patients with advanced-stage CKD. In fact, a decrease of blood urea nitrogen is the most evident effect, likely reflecting a reduction of other nitrogenous molecules not routinely assayed in the clinical practice.

Urea is the main end-product of protein and amino acid metabolism; it is produced by the liver and is finally eliminated into the urine. Approximately 20–30% of urea is hydrolyzed by bacterial urease in the gut with production of ammonia, which may represent a nitrogen source for microbial protein synthesis or can be reabsorbed and made available as a substrate for catabolic or anabolic reactions. Urea metabolism has been widely investigated in humans because it is influenced by physiological and dietetic factors.

Experimental data suggest that urea is toxic at concentrations typical for severe CKD patients [5,54]. Urea directly induces molecular changes related to insulin resistance, apoptosis, and free radical production, and it also damages the gut barrier, as discussed before [54,55]. Serum urea concentrations may be considered as a marker of nitrogen load and retention in CKD, while its role as a cause of clinical manifestation is well known only for very high plasma levels (>200 mg/dL). Urea also generates cyanate, ammonia, and, through cyanate, carbamylated compounds, a non-enzymatic modification of proteins, which have been linked to biological properties [56].

The carbamylation reaction leads to modifications in the protein charge, structure, and function. This process is believed to be involved in accelerated atherosclerosis, increased vascular calcification, and anemia in patients with advanced CKD. Carbamylated compounds may have a potential role in the progression of kidney failure as they take part in the activation of mesangial cells into a cellular intermediate with a profibrogenic action [57].

In CKD patients, the carbamylation reaction may also affect low density lipoproteins which are associated with endothelium cell death, smooth muscle cell proliferation, and monocyte adhesion to endothelium, thus promoting atherogenesis. Carbamylation of high-density lipoproteins also occurs in CKD with the inhibition of endothelial repair mechanisms [58]. Recent clinical studies of protein carbamylation have sought to reveal the unexplained excess risk of morbidity and mortality typical of ESKD patients and have yielded compelling results [59,60,61]. Moreover, small interventional studies have suggested that prolonging dialysis, amino acid supplementation, or a low-protein diet supplemented with KA/EEA may be effective in reducing the carbamylation burden, even though the clinical impact of these measures remain not fully defined.

The carbamylation process peaks at CKD stage 5 to ESKDondialysis transition, and then decreases following dialysis start [62]. Moreover, better survival rates have been observed in patients with the higher degree of carbamylation reduction, independently from traditional risk factors [61].

As already discussed, the increased availability of urea favors cyanate production, a free radical whose levels are increased in CKD. This compound takes part to carbamylation but has no direct toxic effect by itself in promoting endothelial dysfunction [62]; it also affects beta cell glycolysis and insulin secretion with different mechanism respect to that of urea [55].

Despite its well-known metabolic effects which prevent the signs and symptoms of renal failure, delaying the need for dialysis, only a few trials have investigated the effects of LPD and VLPD on gut microbiota and the production of uremic toxins. In this regard, Marzocco et al. compared two groups of patients with CKD that were following two different low-protein diet regimes (standard LPD providing 0.6 g of protein/kg/day versus supplemented VLPD providing 0.3 g of protein/kg/day) [63]. They found more prominent reduction in serum levels of IS in the group that was following the supplemented VLPD in comparison to the LPD group [63]. Accordingly, a more recent study also reported a significant decrease of PCS plasma levels in patients following a LPD in comparison to non-compliant patients [64].

Other promising and more studied approaches have been proposed to improve the intestinal health of patients with CKD/ESKD. More specifically, the use of prebiotics, probiotics, and synbiotics could shift microbial metabolism towards a more saccharolytic direction and reduce the generation of uremic toxins.

To this purpose, the use of prebiotics, which are fermentable fibers that resist gastric acidity and are able to selectively stimulate the growth of saccharolytic bacteria, have been explored in several trials in patients with CKD/ESKD [65,66,67]. This has resulted in major clinical benefits, such as reduction in urea levels [68] and improvement of cardiometabolic and oxidative stress parameters in patients with CKD [69], but also higher fecal nitrogen excretion and increased counts of fecal saccharolytic bacteria [70]. PCS and IS generation rates were also reduced in patients with ESKD that received prebiotic supplementation or fiber-enriched food [67].

Additionally, the use of probiotics was associated to improvements of urea plasma levels and to reduced fecal excretion and reduced serum levels of PCS and IS in patients on hemodialysis [71,72]. Finally, the use of synbiotics (i.e., a combination of probiotics and prebiotics) decreased serum p-cresol conjugates levels and normalized the amount and consistency of stools in HD patients [73]. Overall, both increasing prebiotic fiber and decreasing protein intake seems to result in lowering levels of uremic toxins. Observational data evidenced a positive association between the protein to fiber ratio in predicting the variation of PCS and IS production [74]. However, more studies combining low-protein diets and microbiota manipulation should be performed in order to investigate potential additive effects.

## 4. Once-Weekly Hemodialysis Plus Low-Protein Diet

Keshaviah K. et al. [75] claimed that initiating hemodialysis once a week (OWHD) may be an option; however, it could cause wider swings in the serum concentrations of small-molecular-weight solutes in comparison to schedules based on hemodialysis performed twice a week.

When considering the OWHD frequency, it must be taken into account that the kinetics of urea and of toxic substances of different molecular weight, and the nutritional and metabolic status, significantly change depending on the rhythm and duration of the dialysis sessions. As in the case of conventional thrice-weekly hemodialysis, also when infrequent dialysis rhythms are adopted, urea remains a point of reference for measuring the efficacy of the treatment. In fact, urea is not only an easy marker but it has an own intrinsic direct and indirect toxicity, due to its derivatives such as cyanate and ammonium, which drive protein carbamylation processes [76]. A number of other toxic substances with low MW have the same compartmental distribution, namely guanidines, methyl-guanidine, and malonyl-aldehydes, and they are also easily removed mainly through diffusive dialysis [77]. The evaluation of the weekly Time Average Concentration (TAC) of urea becomes fundamental for the strategic choice of OWHD. Patients with urea TAC of 90 mg/dL showed higher morbidity and mortality than patients with a TAC of 50 mg/dL. Similarly, the TAC of serum methylguanidine was lower in OWHD than in thrice a week HD (mean values 50 vs 65 mcg/dL) [70]. Instead, in regard to the medium-molecules (500–12,000 D) such as β2-microglobulin (β2M), removal by diffusive mechanisms is limited and convective processes are more effective; hence, high-flux hemodiafiltration with high cut-off membranes (possibly with adsorbing characteristics) guarantee a higher removal rate [68]. In the controlled study by Caria et al. [78], after 12 months of observation, pre-dialysis β2M serum levels were stable in OWHD (from 14.2 ± 3.9 to 16.0 ± 5.1 mg/dL), whereas they sharply increased in HD (from 18.4 ± 11.6 to 28.0 ± 11.4 mg/dL, *p* < 0.01). similar results were found in a Japanese cohort of OWHD patients [79]. The preservation of an effective RKF, as it occurs in OWHD regimes, is crucial for obtaining these results.

The scenario changes when dealing with higher molecular weight toxins, especially those strongly bounded to plasma proteins, emblematically represented by the case of Indoxyl-sulphate (IS) and p-cresyl-sulphate (pCS). By applying convective doses with a high exchange of fluids and the use of high porosity membranes, even extending dialysis time to 7–8 h, it is not possible to obtain a relevant removal [80]. In fact, approximately 10% of these molecules, which consists of their ultrafiltrable, not protein-bound fraction, is removed.

Two major factors are relevant in the kinetics of protein-bound uremic toxins in ESKD patients [81]: the first is the conservation and protection of the RKF [82,83], which still allows the removal; the second is the reduced production, obtained by using a lower protein intake and dietary changes inducing modifications of intestinal microbiota. Therefore, the patient in the ESKD stage can enter in a “soft” way into innovative purification strategies in which the transition from the conservative therapy with low-protein diet to the full dialysis dose can be defined as “incremental.” The first step may be represented by an OWHD regimen coupled with LPD in the non-dialysis days. This concept has been well known since the 1980s from the intuitions of Giovannetti et al. [52], subsequently developed by Locatelli et al. with the protocol called Integrated Dialysis Diet Program (IDDP) [84]. Unfortunately, the very limited dietary intake (0.3–0.4 g/ kg/day), supplemented with essential amino acids and ketoacids, caused concerns for increased risk of malnutrition and poor adherence. Twenty years later, Caria et al. proposed the Combined Diet Dialysis Program (CDDP) [78]. The recruited patients had a GFR of 5–10 mL/min/1.73 m^2^, a nutritional prescription of not less than 0.6 g/kg of protein for six days, and a free protein diet on the day of the only weekly dialysis session to compensate for the protein and amino acids losses during the HD session, and a high-efficient hemodialysis (eqKt/V > 1.2). On CDDP, it is not advisable to use total equivalent renal clearance performed by EKR and/or standard clearance (std/KtV), considering urea as the only marker, to target dialysis dose. In fact, EKR is a measure of “downstream” depuration capacity but it does not include the role of low-protein diet, which is an “upstream” depuration. It is preferable to determine the RKF using the average between the residual clearance of urea and that of creatinine (Kr_UREA_ + Kr_sCr_)/2. In the case of values <3 mL/min/1.73 m^2^, the RKF must be accompanied and guaranteed by a two-compartment eqKt/V, in any case always greater than 1.2.

The model represented by the CDDP is based on a multi-compartmental distribution of urea and the evaluation of dietary adequacy makes use of the evaluation of Urea Nitrogen Appearance [50,85] and the periodic evaluation of RKF in order to adjust the dialysis dose according to the changes of RKF over time. The studies available to date regarding OWHD plus protein restriction are summarized in Table 1.

Key parameters are represented by urea generation and the maintenance of a weekly TAC of urea <60–70 mg/dL. These parameters are in fact strongly influenced by dietary intake, intra- and post-dialysis catabolism. For OWHD, it is essential to apply Urea Nitrogen Appearance (UNA) to establish dietary adherence, which is essential to keep patients in a OWHD regimen. UNA takes into account the generation of urea influenced by the dialysis efficacy, by the extent of intra- and post-dialysis catabolism that ends a few hours after the end of the treatment [86,87]. Thus, it should be taken into account the fecal nitrogen output, the accumulation of urea in its volume of distribution (total body water), and finally, the amount of nitrogen eliminated through residual urine output. To verify these parameters and evaluate RKF more realistically, it has been necessary to modify the algorithm called “solute solver,” since this did not fit well with patients with a weekly diuresis of 8–15 L [86,87].

In patients with preserved urine output (>700 mL/day), a significant excretion of phosphorus could be obtained thanks to FGF23-induced lowering of the tubular excretion threshold of phosphorus [88]. The reduced intake coupled with the native kidney output can explain neutral phosphate balance in OWHD. The OWHD regimen also allows better anemia control and saving of erythropoiesis stimulating agents: this is presumably an indirect sign of lower uremic intoxication level [78,79]. In fact, at the same hemoglobin kevels, the EPO dose was much lower in CDDP than in HD control patients. Hypertension and fluid status was well controlled, also thanks to the residual urine volume output. No signs of protein malnutrition were detected and serum albumin was higher in CDDP than in control patients on thrice a week hemodialysis [79]. In addition, an average GFR loss of 1.5 mL/min/1.73 m^2^ per year has been reported in the CDDP group, versus a complete loss of RKF in just three months occurring in patients on three-weekly hemodialysis. Calculation of RKF by the average of creatinine and urea clearance in the last day before dialysis is nearly equivalent to the clearance assessment obtained by urine collection over the six days [87], and it makes easier the close monitoring of RKF.

Besides good adherence to protein restriction, the CDDP requires a major condition, namely substantial urine volume output and optimal volume control. In an ideal CDDP, the weekly hemodialysis should be performed without net ultrafiltration, namely as an iso-volume procedure. This is a crucial point since a very low interdialytic weight gain allows low ultrafiltration rate during the dialysis session, conditions that prevent the accelerated loss of RKF commonly observed in the thrice-a-week schedule and when high ultrafiltration rate must be applied during the dialysis session. Hence, dietary adherence, nutritional and metabolic parameters, and urine volume output (or interdialytic weight gain) should be recorded at least once monthly.

## 5. Conclusions

Lowering uremic toxins is a substantial part of ESKD treatment. Dialysis treatment is a major tool to remove them, but it is not effective for protein-bound or high MW molecules. The removal of these molecules relies, at least in part, on a substantial RKF. Moreover, uremic toxins production is increased in CKD/ESKD, mainly because of the increased protein fermentation by proteolytic bacteria, prevalent in the dysbiotic gut of CKD/ESKD patients. Consequently, a strategy based on the preservation of RKF, together with a low-protein, plant-based diet, may contribute to lower retention of uremic toxins. Unfortunately, both conditions are hardly associated with a thrice-a-week standard dialysis schedule. A combination of a diet restricted in protein, rich in fibers, and consisting of plant-based foods, together with infrequent dialysis therapy, is applicable to stage 5 CKD patients with still-preserved RKF and good attitude to dietary restrictions.. The implementation of the OWHD plus LPD strategy may be useful not only as a gradual, safe, and gentle beginning of dialysis, but also as a tool for lowering uremic toxins.

## Figures and Tables

**Table 1:** Up to date list of the studies existing in the literature that report on the clinical experience data on the combined schedule (once-a-week hemodialysis plus a low-protein diet on the extra-dialysis days) for end-stage kidney disease patients.

Reference	Intervention (No. Patients)	Type of Study	Outcome	Findings
Morelli E et al. 1987 [70]	OWHD+VLPD (17) vs. Control MHD (8)	Prospective controlled non randomized	Clinical findings and blood chemical abnormalities	Reduction of TAC of urea, phosphate and methylguanidine serum levels
Locatelli F et al, 1994 [84]	OWHD+VLPD (84)	Open Cohort Prospective	Nutritional, metabolic, and depurative adequacy.	Good metabolic and depurative adequacy; concerns about dietary adherence, nutritional status and neurological aspects
Caria S et al. 2014 [78]	OWHD+LPD (38) vs. Control MHD (30)	Prospective controlled non randomized	Protection of RKF, nutritional, metabolic-depurative Adequacy	Better preservation of RKF and urine volume and lower serum levels of phosphate, urea, β2-Microglobulin, and ERI; cost saving
Nakao et al. 2018 [79]	OWHD+LPD (112) vs. Control MHD (30)	Prospective controlled non randomized	Protection of RKF, nutritional- metabolic-depurative Adequacy, costs	Better preservation of RKF and urine volume, lower serum levels of phosphorus, urea, β2-microglobulin, and ERI; cost saving

OWHD: once-a-week hemodialysis; VLPD: very low-protein diet supplemented with essential amino acids and ketoacids; MHD: maintenance hemodialysis (thrice-a-week dialysis); LPD: standard low-protein diet; ERI: Erythropoietin resistance index; RKF: residual kidney function.

## Data Availability

Not applicable.
